# Radiomics-Based Detection of Radionecrosis Using Harmonized Multiparametric MRI

**DOI:** 10.3390/cancers14020286

**Published:** 2022-01-07

**Authors:** Clément Acquitter, Lucie Piram, Umberto Sabatini, Julia Gilhodes, Elizabeth Moyal Cohen-Jonathan, Soleakhena Ken, Benjamin Lemasson

**Affiliations:** 1 Univ. Grenoble Alpes, Inserm, U1216, Grenoble Institut Neurosciences, 38000 Grenoble, France; 2 Radiotherapy Department, University Institute Cancer Toulouse Oncopole, 31100 Toulouse, France; Piram.Lucie@iuct-oncopole.fr (L.P.); Moyal.Elizabeth@iuct-oncopole.fr (E.M.C.-J.); 3 INSERM U1037, Team 11, Cancer Research Center of Toulouse (CRCT), 31100 Toulouse, France; Ken.Soleakhena@iuct-oncopole.fr; 4 Institute of Neuroradiology, University Magna Graecia, 88100 Catanzaro, Italy; sabatini@unicz.it; 5 Biostatistics Department, University Institute Cancer Toulouse Oncopole, 31100 Toulouse, France; Gilhodes.Julia@iuct-oncopole.fr; 6 Engineering and Medical Physics Department, University Institute Cancer Toulouse Oncopole, 31100 Toulouse, France

**Keywords:** radiomics analysis, multicenter harmonization, multiparametric MRI, radiation-induced necrosis

## Abstract

**Simple Summary:**

Within a multicentric clinical trial context for the treatment of recurrent high-grade brain tumors, the aim of our study was to assess the potential added value of multiparametric MRI harmonization to improve a classification problem based on radiomics analysis. We confirmed that harmonization reduced the so-called “scanner effect” related to the variability of multiparametric MRI protocol settings between the participating centers and improved the predictive performance of radiomics-based classification model. Radiomics features extracted from MRI perfusion gave the best accuracy for the classification between radionecrosis and tumor progression. Interestingly, our study revealed that radiomics features extracted from T1-weigthed MRI alone, before any injection of contrast product reached accuracies close to the perfusion model.

**Abstract:**

In this study, a radiomics analysis was conducted to provide insights into the differentiation of radionecrosis and tumor progression in multiparametric MRI in the context of a multicentric clinical trial. First, the sensitivity of radiomic features to the unwanted variability caused by different protocol settings was assessed for each modality. Then, the ability of image normalization and ComBat-based harmonization to reduce the scanner-related variability was evaluated. Finally, the performances of several radiomic models dedicated to the classification of MRI examinations were measured. Our results showed that using radiomic models trained on harmonized data achieved better predictive performance for the investigated clinical outcome (balanced accuracy of 0.61 with the model based on raw data and 0.72 with ComBat harmonization). A comparison of several models based on information extracted from different MR modalities showed that the best classification accuracy was achieved with a model based on MR perfusion features in conjunction with clinical observation (balanced accuracy of 0.76 using LASSO feature selection and a Random Forest classifier). Although multimodality did not provide additional benefit in predictive power, the model based on T1-weighted MRI before injection provided an accuracy close to the performance achieved with perfusion.

## 1. Introduction

Glioblastoma is the most frequent and aggressive primary brain tumor in adults. The conventional treatment for *de novo* glioblastoma (GBM) consists of surgery, as complete as possible, followed by radiotherapy with concomitant and adjuvant chemotherapy [1]. Despite treatments, relapse always occurs, leading to a poor outcome for the patient. No consensus on the course of salvage treatment has yet emerged, but whenever the patient’s clinical condition and tumoral extension allow it, local therapies such as second surgery are favored [2]. Among new treatments, hypofractionated stereotactic radiotherapy (hFSRT) presents a solid potential for treating a radio-resistant brain tumor. On recurrent GBM, several regimens of stereotactic radiotherapy showed promising results with acceptable toxicity [3]. However, a possible side-effect of hFSRT is the appearance of radiation-induced brain necrosis sometimes associated with neurological deficiencies [4]. It has also been pointed out that the risk of radionecrosis increases with low fractionation, high dose gradient, and in cases of re-irradiation [5].

In the context of the follow-up of GBMs, radiologists use conventional anatomical MR images (T1w, contrast-enhancement T1w (CE-T1w), T2w, and FLAIR) in addition to more advanced images among which are the perfusion (cerebral blood volume, CBV) and the diffusion (apparent coefficient of diffusion of water, ADC). Such a multiparametric MRI protocol is the technique of choice to evaluate treatment response. While several studies have demonstrated that MR perfusion images are useful for differentiating the radiation-induced necrosis sometimes occurring after radiotherapy or re-irradiation, and all the more so with stereotactic RT, visual appraisal, even by an expert, remains unreliable [6,7,8,9]. The development of an automatic tool to differentiate radiation-induced images from tumor progression has become more important, especially for patients accumulating the risk of brain radionecrosis from both hFSRT and re-irradiation.

Recently, radiomics analysis has been shown to provide useful insights for decision-making in patients bearing GBM under therapies [10,11,12,13]. While neuroradiologists routinely use perfusion and diffusion images to identify radionecrosis, none of these studies investigated such sequences, instead using anatomical images only (CE-T1w and/or FLAIR images) to detect radionecrosis automatically. Most relied on either small cohorts and/or monocentric data, which is one of the main causes of the lack of generalizability of radiomics models. Generalizability refers to the ability of a model trained on a given dataset to be successfully applied to a different population. A possible approach to tackling this shortcoming and therefore increasing the sensitivity of the study is to feed the radiomics model with features extracted from a multicentric dataset. Unfortunately, pooling data acquired from different centers induces variability related to the scanner’s properties or settings (imaging protocol, reconstruction algorithm), which in turn affects the robustness of the radiomics feature. This phenomenon, also called the “scanner effect”, creates a bias independent of the biological properties [14] that may lower the efficiency of predictive models [15]. To overcome this effect, multicenter harmonization algorithms have been developed to remove the “scanner effect” while preserving the biological properties of images. In recent research, a Bayesian method called ComBat [16] was successfully applied to remove the variability from imaging features extracted from different modalities [17]. ComBat has been studied on both phantom and clinical data to demonstrate its effectiveness in counterbalancing the scanner effect on the radiomic features extracted from images [15]. However, to the best of our knowledge, no study has been done to evaluate the impact of multicenter harmonization on a classification task such as the detection of radionecrosis.

The aim of this study was two-fold: first, the sensitivity of radiomic features extracted from multiparametric MRI to scanner-related effects was identified and characterized. Moreover, the ability of ComBat harmonization to remove these non-biological variations was evaluated. In a second phase, the problem of differentiating radionecrosis from tumor recurrence via radiomics analysis was investigated. In particular, the performances of models trained with raw versus harmonized features were compared. Finally, the added value of incorporating diffusion and perfusion MRI into radiomics models was assessed and compared with the use of anatomical images only.

## 2. Materials and Methods

### 2.1. Patient Population

The study was conducted according to the guidelines of the Declaration of Helsinki and approved by the Institutional Review Board of Comité de Protection des Personnes Sud-Ouest et Outre-Mer III (CPP SOOM III) under the number 2016/62 on 27 July 2016. Data were collected as part of the ongoing multicentric clinical trial STERIMGLI (NCT02866747) [18]. For this study, the patient population was composed of 28 subjects divided into two arms: the patients in arm A (*n* = 5) had hFSRT alone, while the subjects included in arm B received both hFSRT and anti-PDL1 immunotherapy (Table 1). The multiparametric MRI protocol included the acquisition of anatomical images (T1w, CE-T1w, T2w, and FLAIR), diffusion (DWI), and perfusion (DSC) MRI. This protocol was applied to each patient during the screening phase and every eight weeks until a local progression was observed. A total of 102 multiparametric MRI examinations acquired in five centers using seven different scanners were used in this study. Radionecrosis was established based on RANO and iRANO criteria [19] for each longitudinal conventional MRI acquisition by the local radiologist of the participating center. In addition, a centralized expert review by two specialists, a radiation oncologist (L.P.) and a neuro-radiologist (U.S.) included advanced techniques such as perfusion and diffusion for the quantitative analysis, to identify or exclude the presence of radionecrosis for each MRI session.

### 2.2. Images Preprocessing

Anatomic images were preprocessed according to the pipeline provided by the BraTS toolkit [20] (https://github.com/neuronflow/BraTS-Toolkit, last accessed on 4 December 2021). Briefly, all anatomical modalities were first co-registered to the T1 space of the patient. The brain mask was extracted with HD-BET [21], and a registration to a space common to all patients (BraTS space) was finally performed. The registration steps were carried out using the Advanced Normalization Tools (ANTs). Apparent diffusion coefficient (ADC) maps were derived from diffusion-weighted MR images (DWI) using MRtrix [22]. We applied the function dwi2adc, implemented in MRtrix, directly on the DWI images, then the resulting ADC images were registered to the BraTS space using ANTs. The relative cerebral blood volume maps were computed from the DSC-MRI examination using Olea Sphere version 3.0 SP7 (Olea Medical). The pipeline implemented for CBV analysis included motion correction, deconvolution with block-circulant singular value decomposition, and permeability correction. For the sake of clarity, in the rest of the document, apparent diffusion coefficient maps and relative cerebral blood volume maps will be called diffusion and perfusion maps, respectively. Diffusion and perfusion maps were then co-registered to anatomical images in the BraTS space with ANTs. Intensity normalization was performed for all anatomical images (T1w, CE-T1w, T2w, and FLAIR) using the WhiteStripe (WS) method [23] implemented in the Python library found at https://github.com/jcreinhold/intensity-normalization (last accessed on 4 December 2021) [24]. The normalization was performed on the preprocessed images, i.e., on the images co-registered in the BraTS space. To identify and characterize the scanner effect on radiomic features, we drew a spherical region-of-interest (ROI) in the center of the brain called the healthy ROI. This region, common to all patients, was chosen so that the tissue appeared homogeneous across patients (i.e., outside the pathological area and with low biological variation) to quantify the variation caused by the multicenter effect. Then, for the radionecrosis detection, we defined a pathological ROI consisting of enhanced lesions on CE-T1w images. For this purpose, we first performed a multiclass segmentation of the tumor using the BraTS toolkit [20]. Then, the pathological ROI was revised by two clinicians (a junior radiation-oncologist and a senior neuro-radiologist) and, if necessary, manually corrected [25].

### 2.3. Feature Processing

Radiomic features of the pathological ROI were extracted from each MR image (T1w, CE-T1w, T2w, FLAIR, diffusion, and perfusion) using the pyRadiomics framework [26]. A total of 600 features corresponding to 100 features/MR images were computed, corresponding to the first-order statistics, texture, and shape-based features. To ensure a better reproducibility of features, gray-level discretization was performed with a fixed bin width. No other preprocessing was executed during feature extraction as images had already been resampled and normalized earlier in the pipeline. To take into account the imbalanced distribution across classes, minority class oversampling was applied using the synthetic minority oversampling technique (SMOTE, as found at https://imbalanced-learn.org, last accessed on 4 December 2021). The multicenter harmonization was performed using the ComBat algorithm, a batch-effect correction tool initially developed for genomics data [17]. The Python implementation found at https://github.com/Jfortin1/ComBatHarmonization (last accessed on 4 December 2021) was used with the parametric prior method in the empirical Bayes procedure. The harmonization was performed at the feature level, and radiomics vectors corresponding to each radiomic extracted from one MR image were harmonized independently. The radionecrosis status was used as a covariate to be preserved during the harmonization process.

### 2.4. Radiomics Modeling

**Figure 1:**
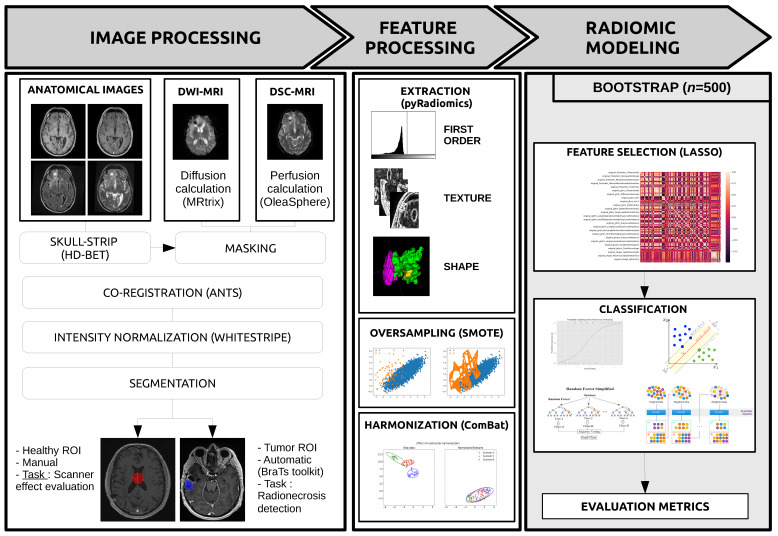
Radiomics pipeline.

The classification problem evaluated in this work consisted in the detection of radionecrosis in multiparametric MRI. The clinical outcome was extracted from the case report form (CRF) used to collect data from each participating hospital. Two sets of experiments were considered to assess the impact of harmonization on the predictive power of radiomics modeling. First, radiomic models were trained on raw features without harmonization and compared to models based on features harmonized with ComBat. Second, feature selection was carried out using the least absolute shrinkage and selection operator (LASSO) method [27]. All features with non-zero importance coefficients were retained for model building. The classification problem was solved with four algorithms: Logistic Regression (LR), Support Vector Machine (SVC), Random Forest (RF), and AdaBoost (AD). Performance metrics (balanced accuracy, sensitivity, and specificity) were computed using a bootstrap approach where the original dataset was resampled with replacement 500 times into training and testing sets with ratios of 2/3 and 1/3 respectively. SMOTE [28] was applied to the training set only, to compensate for the imbalanced dataset. A Wilcoxon signed-rank test was performed to determine if there was a statistically significant difference in the balanced accuracy before and after ComBat. The null hypothesis that the two distributions had no difference was rejected for *p*-values < 0.001. The whole process is illustrated in Figure 1.

## 3. Results

Our results are divided into two parts. The first part is devoted to heterogeneity characterization and correction: we characterized the presence of variability in MR acquisition parameters and radiomic features from the healthy ROI. The second part aims to assess the added value of perfusion and diffusion to differentiate radionecrosis from tumor progression and the impact of heterogeneity correction on the prediction.

### 3.1. Characterization and Correction of the “Scanner Effects”

**Figure 2:**
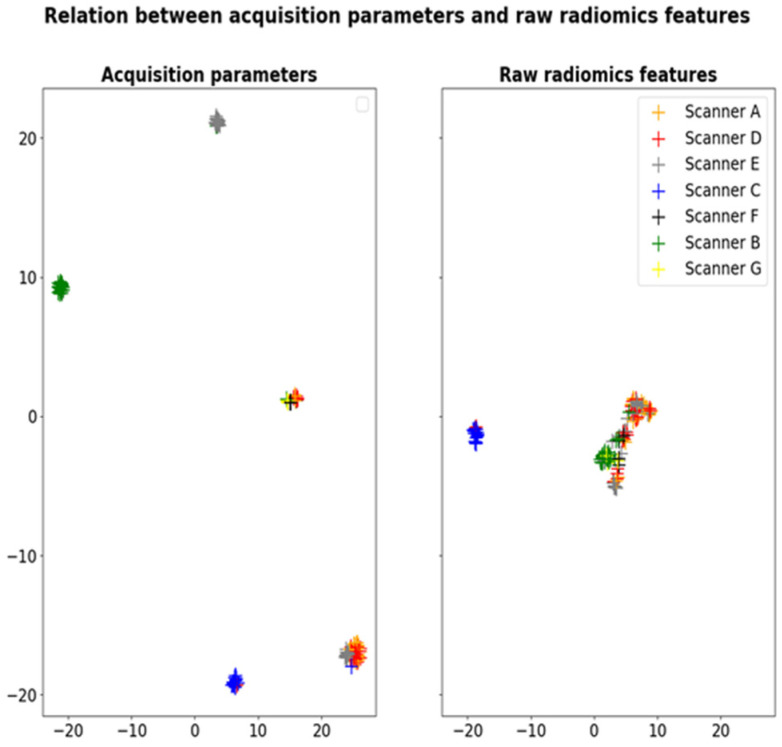
Dimensionality reduction of acquisition parameters TE, TR, flip angle, slice thickness and pixel spacing (left) and radiomics features extracted from the healthy ROIs in T1w images (right) with UMAP showing the sensitivity of radiomic features to the scanner effects.

Although the dataset originated from a coordinated clinical trial, several protocols were used by the different participating centers, leading to significant variability in MRI acquisition parameters (Table 2). For example, in anatomical T1w images, the echo time ranged from 2.4 to 8.5 ms, and the repetition time was between 7.6 and 2080 ms. This variability in T1w acquisition parameters (TE, TR, flip angle, slice thickness, and pixel spacing) is illustrated as UMAP clusters in Figure 2. Thus, one can observe several groups of acquisition parameters corresponding roughly to different participants’ MRI scanners. Interestingly, the same clusters are observed when applying UMAP dimensionality reduction to the radiomics features extracted from the healthy ROIs (cf. Figure 2). These results demonstrate the sensitivity of radiomic features to the variability in MRI acquisition protocols.

**Figure 3:**
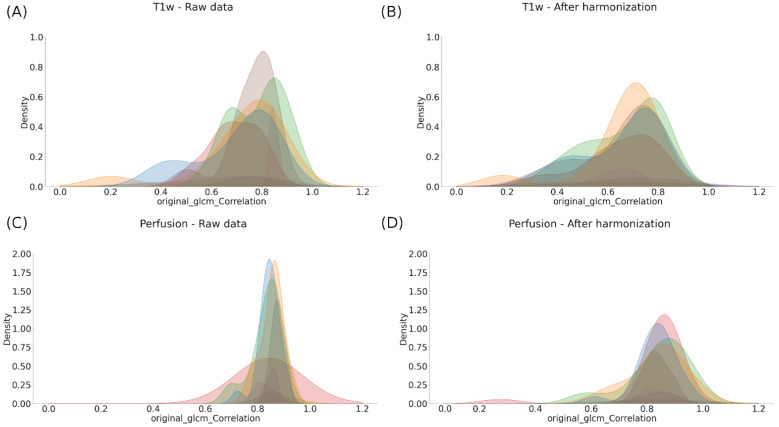
Effect of harmonization on the distribution of an example radiomic feature extracted from the healthy ROIs across the different scanners (namely, glcm-Correlation) before (**A**,**C**) and after ComBat harmonization (**B**,**D**).

**Figure 4:**
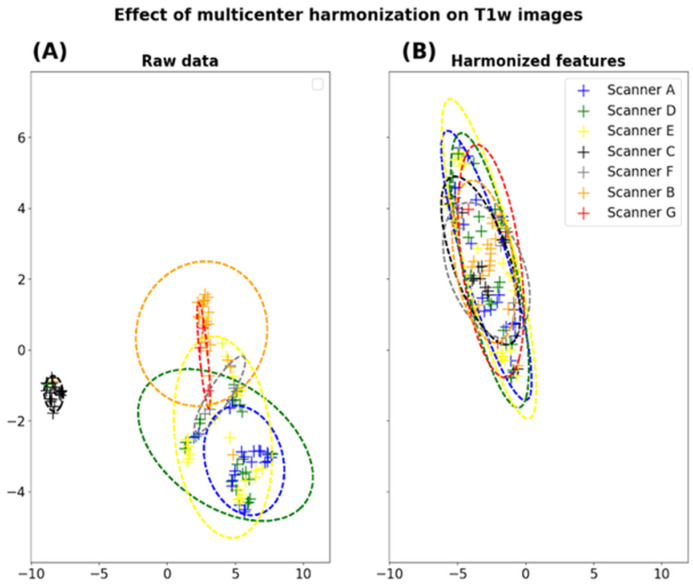

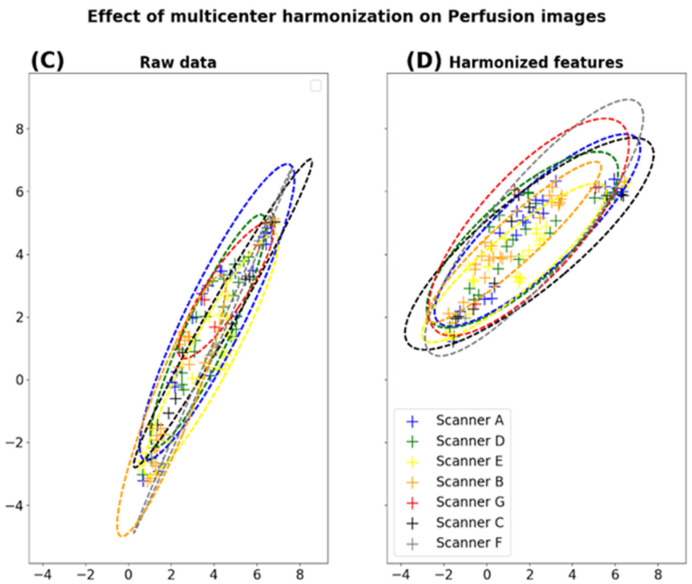
Dimension reduction of radiomic features extracted from the healthy ROIs on T1w and perfusion images. For each modality, a UMAP clustering is performed on raw data (**A**,**C**) and after harmonization (**B**,**D**).

To compensate for the “scanner effects” observed in our dataset, we evaluated the impact of image normalization using WS combined with ComBat harmonization. One can observe the impact of this method at the feature scale using histogram-based representations on one representative feature (Figure 3) and for all radiomic features using the UMAP representations (Figure 4). 

Figure 3A,B shows the contribution of the normalization of anatomical images followed by the harmonization of the metrics extracted to the homogeneity of our cohort (here illustrated using T1w). Indeed, it is observed that the histograms of a texture radiomics feature (namely glcm-Correlation) are found to be superimposed after these two stages of compensation, compared to the raw data (Figure 3). In addition to the classical anatomical MR protocol, our protocol included diffusion and perfusion images, which are known to be more quantitative than anatomical MRI. As the intensity normalization is already included in the calculation of the diffusion and perfusion maps, we only investigated the impact of ComBat harmonization on these scans. Here, too, although still imperfect, a beneficial effect on the harmonization of the perfusion and diffusion images using the Combat algorithm was observed (Figure 3C,D).

### 3.2. Prediction of the Radionecrosis

**Figure 5:**
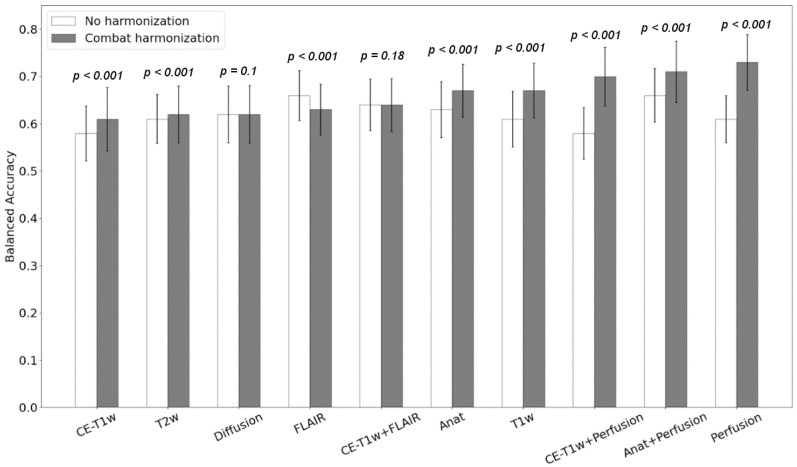
Classification accuracy obtained with the Logistic Regression model before (white) and after ComBat harmonization (gray). The *p*-values from the Wilcoxon test are shown for each pair. The x-axis refers to radiomics models based on features extracted from different combination of modalities. Anat refer to the radiomic model based on anatomical MR images (T1w, CE-T1w, T2w and FLAIR).

First, we observed that all classification scores were improved when the “scanner effects” were compensated, except for the model using FLAIR features only (balanced accuracy of 0.66 vs. 0.63, sensitivity of 0.65 vs. 0.64, and specificity of 0.66 vs. 0.62, respectively, without harmonization, and 0.63 with ComBat harmonization). The most significant increase in the radionecrosis prediction was observed for the model based on perfusion features only (balanced accuracy 0.61 vs. 0.73, sensitivity 0.6 vs. 0.75, and specificity 0.61 vs. 0.78, respectively) (Figure 5).

Interestingly, our results show that classification models trained on multiparametric radiomic features do not necessarily outperform simpler models based on a single MR modality such as perfusion.

Table 3 presents the balanced accuracy of each classifier obtained with two models (perfusion-based and T1w-based models) with or without “scanner effects” correction. These results show that the investigated models are robust with respect to the choice of classification method.

### 3.3. Radiomics Signature

**Figure 6:**
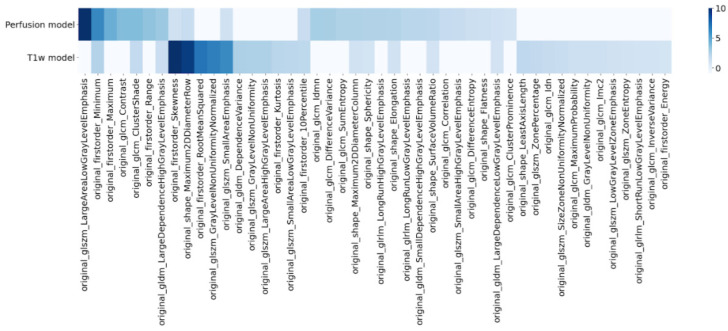
Selected features for the two best radiomics models dedicated to the detection of radionecrosis: perfusion-based model (top) and T1w-based model (bottom).

A radiomics signature was extracted from the radiomic models based on T1w and perfusion images. These signatures are based on the importance coefficients of the LASSO feature selection step. For each model, the mean value of the importance coefficients above the bootstrap runs was computed. All radiomics features with a mean importance above 1% are shown in Figure 6. For the perfusion model, the most important feature in the signature is based on the gray level size zone texture matrix (namely glszm_LargeAreaLowGrayLevelEmphasis). In this model, minimum and maximum intensity and intensity range are also considered relevant to discriminating between radionecrosis and tumor progression. 

Regarding the T1w-based model, the most important selected features were the first order skewness metric, a shape feature, closely followed by several textural features.

## 4. Discussion

This study aimed to describe the “scanner effect” in the different modalities acquired in a multiparametric and multicenter study. We evaluated the impact of the radiomic features harmonization on a multicentric MRI dataset to differentiate radionecrosis from tumor recurrence. This evaluation was performed using a conventional anatomical MR protocol and more advanced MR images such as perfusion and diffusion maps.

Our study provides interesting insights into the research topics investigated. First, our results provided additional evidence regarding the sensitivity of radiomic features to the variability of imaging protocols in multicentric MRI studies. This non-biological variability was observed on radiomics features extracted from each MR modality (T1w, T2w, FLAIR, diffusion, and perfusion) investigated in this study. Previous work has already reported such sensitivity for anatomical MRI [15] and diffusion [19] maps but, to the best of our knowledge, it is the first time that the multicenter variability has been described for data derived from perfusion MRI (CBV).

Secondly, we have shown the ability of ComBat harmonization to remove non-biological variability in radiomic features extracted from MR images. Our results strengthen other findings, as the benefits of feature harmonization have already been pointed out in [15,19]. In particular, Orlhac et al. demonstrated that multicenter harmonization using a combination of image standardization (with WhiteStripe) and ComBat realignment could remove protocol-based variations in structural MR images (T1w and FLAIR images) acquired in two different centers. Our study used MRI data originating from five centers and acquired on seven different machines, thus expanding the scope of the considered methods to the scale of a real-world clinical trial. Additionally, the presence of a scanner effect and the impact of ComBat harmonization on radiomic features extracted from diffusion and perfusion MRI were also assessed. In both cases, the use of ComBat harmonization allowed us to compensate the non-biological variability in radiomic features.

Finally, with unharmonized radiomic features, the best classification performance was obtained with the FLAIR model. This result is difficult to interpret as radiological properties in FLAIR MRI are supposed to be similar for recurrent tumor and radiation necrosis. After harmonization, however, the best model stemmed from the perfusion radiomics feature, as expected. Indeed, radionecrosis lesions include vascular injury, translating into a hypoperfusion, as opposed to tumor recurrence which is associated with a neoangiogenesis and therefore hyperperfusion. In Barajas et al., the authors showed that perfusion MRI was significantly higher within the recurrent GBM patients than within the radionecrosis group [9]. The results presented in [7] also show that perfusion MRI maps (both absolute and relative CBV) were efficient in the differentiation of radionecrosis and tumor recurrence after irradiation.

Other studies also showed the ability of MRI-based radiomics analysis to discriminate between true tumor progression and radiation-induced necrosis. In [19], the authors used radiomic features extracted from CE-T1w images to distinguish between radionecrosis and progression with an accuracy of 0.75. In [12], CE-T1w and FLAIR MRI features were extracted for differentiating between treatment effect and true progression, with a sensitivity of 0.65. In our study, radiomic models based on CE-T1w and FLAIR features were not the best performing, especially after ComBat harmonization. This result seems more consistent with clinical observations since conventional radiological features based on CE-T1w and FLAIR MRI have been shown to have low sensitivity in discriminating radionecrosis from recurrence [29,30]. In [13], several radiomic models based on CE-T1w, T2, and diffusion images were investigated to differentiate radionecrosis from tumor progression in patients treated with radiotherapy. In the study by Park et al., the model with the best discriminating performance was based on diffusion images. Combining both anatomical and diffusion-based radiomic features into the same model improved the performance. However, in our study, the diffusion-based model produced the worst performance. Another study pointed out that the ComBat method showed some limits for multi-site diffusion MRI [31], and further investigation should be carried out to clarify this point.

Another interesting result is the good accuracy reached by the T1w-based model (without contrast agent). Our study found that T1w images provided a better classification score than T1 post-contrast images. This result could be explained by the heterogeneity observed at low signal intensity for T1w MRI that are rarely interpreted by neuro-radiologists. The classification model trained with perfusion radiomic features showed improved performance when used with ComBat harmonization. This result is consistent with other results found in the literature [7,9,32]. Perfusion can improve the accuracy of differentiating necrosis from a recurrent tumor in patients with brain tumors. 

This study has several limitations. First, the small number of patients included in the experiments may appear insufficient for model validation. We aim to pursue and validate the analysis with the rest of the patients’ data from the ongoing and still recruiting clinical trial. Second, to increase the dataset size, we considered the different sessions of the same patient as independent samples. However, this procedure may insert redundancies and alter the robustness of the performance metrics. Furthermore, although the evaluation of radiomics models was carried out with a bootstrap procedure, no external validation was performed due to the lack of data. In future work, model validation should be considered on an external dataset to improve the study’s statistical power. Moreover, increasing the dataset size would allow a radiomics signature for discriminating radionecrosis from another treatment effect to be validated more robustly. 

## 5. Conclusions

This work aimed to perform a radiomics analysis on multiparametric MRI in the context of a multicentric clinical trial. The main objective was to evaluate the added value of diffusion and perfusion MRI for the computer-aided differentiation of radionecrosis and tumor progression in patients treated with radiotherapy. The identification and characterization of scanner-related variability on radiomic features were performed. This non-biological heterogeneity affected the radiomic features extracted from the different MRI modalities included in the imaging protocol (anatomical, perfusion, and diffusion). Image normalization and feature harmonization with ComBat were found to reduce the scanner effects successfully. These results confirm the results of previous studies, while extending the scope to new modalities such as perfusion MRI.

Moreover, radiomic models based on harmonized features showed improved performance when compared to unharmonized models. In particular, the detection of radionecrosis using radiomics analysis was best achieved with perfusion-based models. A good accuracy approaching the perfusion model performance was achieved with T1w images, allowing the diagnosis of radionecrosis in clinical routine, even when perfusion images are lacking, and therefore improving the evaluation of treatment effects for patients treated with radiotherapy.

## Figures and Tables

**Table 1:** Clinical characteristics of the patient population in the dataset.

Patient Characteristics		Phase I	Phase II	
			Arm A	Arm B
Total		6	6	16
Age (mean = 56)				
Sex	Male	3	4	12
	Female	3	2	4
Surgery	Biopsy	2	1	3
	Near-complete resection	3	4	7
	Complete resection	2	1	9
MGMT Status	Methylated	3	3	11
	Unmethylated	3	3	5
Radionecrosis status	Positive	2	1	9
	Negative	4	5	7

Arm A refers to patients treated with radiotherapy alone and arm B to patients treated with radiotherapy and immunotherapy.

**Table 2:** Characteristics of each MRI protocol.

		A	B	C	D	E	F	G
MRI examination		20	10	24	22	23	3	3
Radionecrosis		5	1	10	11	9	1	1
MRI Model		Siemens Aera	GE Optima	Siemens Skyra	Siemens Aera	Siemens Skyra	GE Optima	GE Signa
Magnetic Field		1.5	1.5	3	1.5	3	1.5	3
T1w	TE (ms)	11.0	7.6	220	11.0	2200	7.63	600
	TR (ms)	5.37	3.16	2.49	5.37	2.48	3.1	10.4
	FA (°)	15	15	70	15	8	15	90
T2w	TE (ms)	7540	6000	800	8250	5300	81	58
	TR (ms)	115	100	20	115	111	48.5	30
	FA (°)	170	160	20	170	150	30	15
FLAIR	TE (ms)	7000	12,000	8000	7000	6600	8000	9800
	TR (ms)	124	131.3	140	124	349	123.3	141
	FA (°)	180	160	150	180	120	90	90
DWI	TE (ms)	7800	8000	6430	7800	7110	4500	11,700
	TR (ms)	70	72.4	71	107	64	69.9	72.7
	FA (°)	180	90	180	90	180	90	90
DSC	TE (ms)	1880	1800	1980	1970	1770	2000	1770
	TR (ms)	30	65	30	30	25	60	25
	FA (°)	90	90	90	90	90	90	90

Echo time (TE) and repetition time (TR) are given in milliseconds (ms) and flip angle (FA) in degrees (°). A to G refer to the identification letters assigned to the different MRI scanners in this study. Anatomical images: T1-weighted (T1w), T2-weighted (T2w) and Fluid-attenuated Inversion Recovery (FLAIR); diffusion: Diffusion Weighted Imaging (DWI); perfusion: Dynamic Susceptibility Contrast (DSC).

**Table 3:** Classification scores (balanced accuracy, sensitivity, and specificity) before and after harmonization for the two reference models (perfusion-based and T1w-based models).

Classification Score		Perfusion		T1w	
		Non-ComBat	ComBat	Non-ComBat	ComBat
Logistic Regression	B. Accuracy	0.61 ± 0.05	0.73 ± 0.059 (*)	0.61 ± 0.059	0.67 ± 0.058 (*)
	Sensitivity	0.6 ± 0.109	0.75 ± 0.09	0.6 ± 0.11	0.65 ± 0.108
	Specificity	0.61 ± 0.105	0.7 ± 0.101	0.63 ± 0.117	0.68 ± 0.097
Support Vector Classifier	B. Accuracy	0.6 ± 0.057	0.72 ± 0.057 (*)	0.61 ± 0.059	0.66 ± 0.062 (*)
	Sensitivity	0.62 ± 0.126	0.73 ± 0.094	0.61 ± 0.109	0.65 ± 0.12
	Specificity	0.59 ± 0.115	0.71 ± 0.107	0.62 ± 0.118	0.67 ± 0.106
Random Forest	B. Accuracy	0.63 ± 0.052	0.75 ± 0.06 (*)	0.6 ± 0.059	0.64 ± 0.056 (*)
	Sensitivity	0.63 ± 0.126	0.75 ± 0.107	0.57 ± 0.13	0.63 ± 0.127
	Specificity	0.64 ± 0.121	0.76 ± 0.109	0.62 ± 0.142	0.65 ± 0.124
AdaBoost	B. Accuracy	0.6 ± 0.059	0.76 ± 0.063 (*)	0.58 ± 0.062	0.61 ± 0.063 (*)
	Sensitivity	0.6 ± 0.112	0.76 ± 0.102	0.57 ± 0.13	0.61 ± 0.114
	Specificity	0.6 ± 0.115	0.76 ± 0.102	0.59 ± 0.116	0.62 ± 0.119

(*) refers to significant differences in balanced accuracies. B. accuracy refers to balanced accuracy.

## Data Availability

The data presented in this study are available on request from the corresponding author. The data are not publicly available due to the unfinished clinical trial.

## Abbreviations

The following abbreviations are used in this manuscript:

MRI	Magnetic Resonance Imaging
GBM	Glioblastoma
hFSRT	Hypofractionated Stereotactic Radiotherapy
CE-T1w	Contrast-Enhancement T1w
CBV	Cerebral Blood Volume
ADC	Apparent Diffusion Coefficient
DWI	Diffusion-Weighted Imaging
DSC	Dynamic Susceptibility Contrast
ROI	Region of Interest
SMOTE	Synthetic Minority Oversampling Technique
CRF	Case Report Form
LASSO	Least Absolute Shrinkage and Selection Operator
LR	Logistic Regression
SVC	Support Vector Classifier
RF	Random Forest
AD	AdaBoost
WS	WhiteStripe
